# Dual IL-6 and CTLA-4 blockade regresses pancreatic tumors in a T cell– and CXCR3-dependent manner

**DOI:** 10.1172/jci.insight.155006

**Published:** 2023-03-07

**Authors:** Michael Brandon Ware, Maggie Phillips, Christopher McQuinn, Mohammad Y. Zaidi, Hannah M. Knochelmann, Emily Greene, Brian Robinson, Cameron J. Herting, Thomas A. Mace, Zhengjia Chen, Chao Zhang, Matthew R. Farren, Amanda N. Ruggieri, Jacob S. Bowers, Reena Shakya, Alton B. Farris, Gregory Young, William E. Carson, Bassel El-Rayes, Chrystal M. Paulos, Gregory B. Lesinski

**Affiliations:** 1 Department of Hematology and Medical Oncology,; 2 Department of Surgery, Winship Cancer Institute of Emory University, Atlanta, Georgia, USA.; 3 Division of Surgical Oncology, Department of Surgery, Department of Internal Medicine, The Ohio State University, Columbus, Ohio, USA.; 4 Department of Microbiology and Immunology, Hollings Cancer Center, Medical University of South Carolina, Columbia, South Carolina, USA.; 5 Department of Pathology, Winship Cancer Institute of Emory University, Atlanta, Georgia, USA.; 6 Division of Gastroenterology Hepatology and Nutrition, Department of Internal Medicine, The Ohio State University, Columbus, Ohio, USA.; 7 Department of Biostatistics, Emory University, Atlanta, Georgia, USA.; 8 Comprehensive Cancer Center and; 9 Center for Biostatistics, The Ohio State University, Columbus, Ohio, USA.

**Keywords:** Immunology, Oncology, Cancer immunotherapy, T cells

## Abstract

This study aimed to enhance antitumor immune responses to pancreatic cancer via Ab-based blockade of IL-6 and cytotoxic T-lymphocyte–associated protein 4 (CTLA-4). Mice bearing s.c. or orthotopic pancreatic tumors were treated with blocking Abs to IL‑6 and/or CTLA-4. In both tumor models, dual IL-6 and CTLA-4 blockade significantly inhibited tumor growth. Additional investigations revealed that dual therapy induced an overwhelming infiltration of T cells into the tumor as well as changes in CD4^+^ T cell subsets. Dual blockade therapy elicited CD4^+^ T cells to secrete increased IFN-γ in vitro. Likewise, in vitro stimulation of pancreatic tumor cells with IFN-γ profoundly increased tumor cell production of CXCR3-specific chemokines, even in the presence of IL-6. In vivo blockade of CXCR3 prevented orthotopic tumor regression in the presence of the combination treatment, demonstrating a dependence on the CXCR3 axis for antitumor efficacy. Both CD4^+^ and CD8^+^ T cells were required for the antitumor activity of this combination therapy, as their in vivo depletion via Abs impaired outcomes. These data represent the first report to our knowledge of IL-6 and CTLA‑4 blockade as a means to regress pancreatic tumors with defined operative mechanisms of efficacy.

## Introduction

Abs targeting immune checkpoint receptors and their ligands have gained regulatory approval and demonstrated efficacy in patients. The most notable examples include blockade of cytotoxic T-lymphocyte antigen-4 (CTLA-4) and programmed cell death protein 1 (PD-1). Despite encouraging data in patients with various malignancies, there remain a number of key challenges with this approach (1). Many patients still do not benefit from immune checkpoint inhibition (ICI), while resistance is common in those who do initially respond to therapy (1). Most pancreatic tumors are also inherently resistant to ICI (2). Overcoming these limitations is a priority and could advance outcomes across multiple tumor types.

The desmoplastic stroma unique to pancreatic ductal adenocarcinoma (PDAC) is a dynamic, immune-suppressive component contributing to the limited impact of immune therapy in this malignancy. Our laboratory and others have demonstrated that PDAC stroma and stromal-derived cytokines and chemokines restrain host immunity (3–7). Although dysregulated cytokines represent rational targets, there are limited data to prioritize them in patients. In a cohort of 72 treatment-naive patients with metastatic PDAC, IL-6 correlated with reduced overall survival (8). These data were intriguing, as IL-6 can regulate phenotypic and functional properties of various lymphocyte and myeloid cell populations (9–11). Detailed immune phenotyping of peripheral blood cells from the same patient cohort revealed a similar relationship between reduced overall survival and elevated circulating CTLA-4^+^ T cells (8).

These data encourage strategic combination therapies incorporating CTLA-4–targeted Abs in PDAC. Based on these data, we hypothesized that IL-6 blockade would enhance efficacy of anti–CTLA-4 therapy. Herein, we report that the combined blockade of IL-6 and CTLA-4 inhibits PDAC growth by potentiating infiltration of T cells into tumors. Aligned with these observations, we demonstrate that PDAC cells produce lymphocyte-attracting CXCR3-associated chemokines in response to IFN-γ stimulation, including high levels of the CXCR3-associated chemokine CXCL10. In vitro, dual IL-6/CTLA-4 blockade also promotes IFN-γ production by CD4^+^ T cells. In vivo studies further indicate systemic shifts and intratumoral increases in CXCR3-expressing CD4^+^ T cells after dual IL‑6/CTLA-4 blockade and antitumor efficacy that are dependent upon CXCR3. This treatment also increased CD4^+^ Th cell subsets expressing TBET and GATA3 in splenocytes from mice. Finally, this therapy relies on both CD4^+^ and CD8^+^ T cells for its efficacy. Together, our findings suggest that combined blockade of IL-6 and CTLA-4 can regress pancreatic tumors via a potentially unique mechanism by imparting CD4^+^ and CD8^+^ T cell–mediated antitumor immunity.

## Results

### Combined blockade of IL-6 and CTLA-4 augments antitumor efficacy in murine PDAC models.

**Figure 1:**
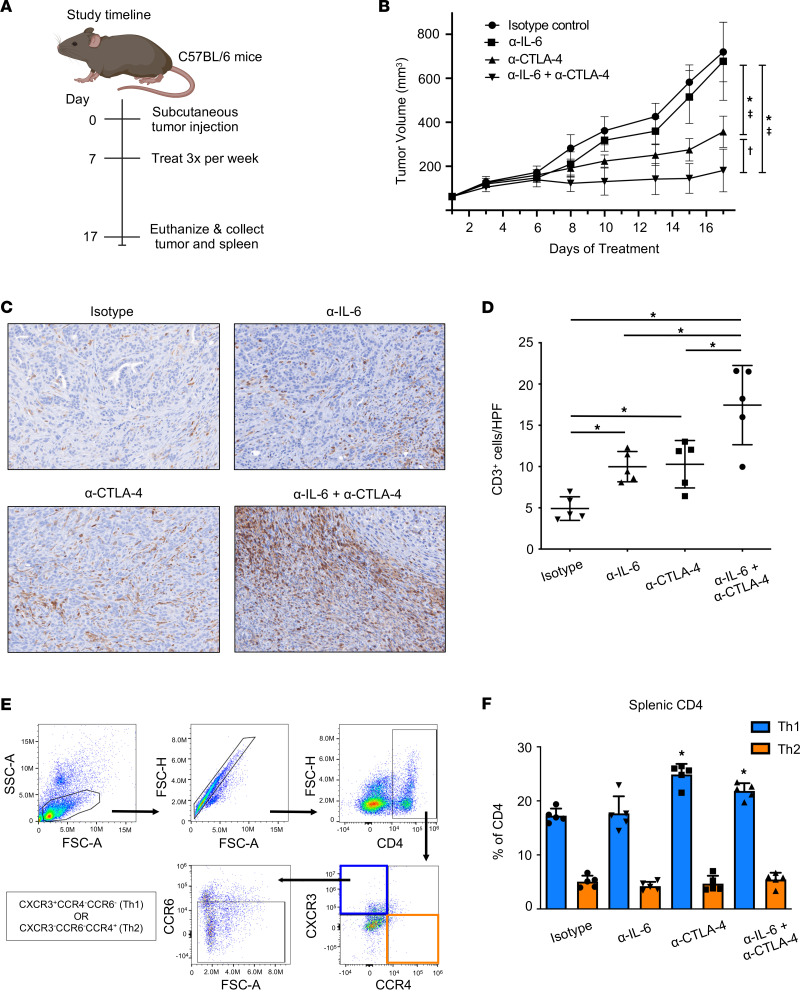
Combined blockade of IL-6 and CTLA-4 significantly inhibits tumor growth and promotes CD3 T cell infiltration of tumors in an s.c. murine model of pancreatic cancer. Study timeline is shown in (**A**). MT5 murine pancreatic tumor cells were s.c. injected into C57BL/6 mice with treatment beginning when tumors reached 50–100 mm^3^. Mice were treated with 200 mg (i.p. injection 3 times/week) of isotype control, cytokine blockade anti–IL-6 and/or anti–CTLA-4 Abs (*n* = 5 mice/group) until mice met prespecified IACUC-approved early removal criteria. (**B**) Changes in tumor volume as determined by caliper measurement throughout the course of Ab treatment. Mean ± SD; *P < 0.05 versus isotype control; ‡*P* < 0.05 versus anti–IL-6; and †*P* < 0.05 versus anti–CTLA-4 using mixed-effects regression. (**C**) Representative 20× images of IHC staining for CD3 in FFPE tumor tissue slices from mice in the different treatment groups. (**D**) Mean ± SD for percent of cells expressing CD3^+^ in s.c. tumors per high-powered field. Symbols represent individual mice; * indicates significance compared with isotype control-treated mice. *P* values for significant comparisons with ANOVA and LSD for pairwise comparisons are as follows: isotype versus α–IL-6 (0.0013); isotype versus α–CTLA-4 (0.0058); and isotype versus α–IL-6 + α–CTLA-4 (0.0058). (**E**) Splenocytes were isolated from the mice receiving treatment as stated in **B**. Flow cytometry was performed with Abs against CD4, CCR6, CXCR3, CCR4, and RORγt. CD4^+^CCR6^–^CXCR3^+^CCR4^–^ were identified as suggestive of a Th1 phenotype and CD4^+^CCR6^–^CXCR3^–^CCR4^+^ as a Th2 phenotype. (**F**) Graph of mean percentages of CD4^+^ T cells that have a Th1 or Th2 phenotype. Data shown as mean ± SD; * indicates significance compared with isotype control-treated mice with ANOVA and LSD for pairwise comparisons. *P* values for significant comparisons are as follows: isotype versus α–CTLA-4 (< 0.0001); isotype versus α–IL-6 + α–CTLA-4 (0.0004); α–IL-6 versus α–CTLA-4 (0.0026); and α–IL-6 versus α–IL-6 + α–CTLA-4 (0.0278).

We first tested our hypothesis that dual IL-6 and CTLA-4 blockade therapy could improve immune responses to pancreatic cancer in mice bearing s.c. MT5 tumors. PDAC tumors were established in mice and then treated with either anti–IL-6 and/or anti–CTLA-4 Abs, as shown in scheme Figure 1A. For these studies, the MT5 cell line was used, which originated from a KPC tumor, harboring G12D-mutated (Kirsten rat sarcoma viral oncogene homologue) *Kras* and R172H-mutated *Trp53* (12). Indeed, we found that tumor growth was significantly reduced in mice treated with combined IL-6– and CTLA‑4–blocking Abs compared with mice treated with isotype control Abs (*P* = 0.0001), single-agent CTLA-4 blockade (*P* = 0.0207), or IL-6 blockade (*P* = 0.0002) (Figure 1B). Although anti–CTLA-4 alone inhibited tumor growth to a greater extent than mice treated with an isotype control (*P* = 0.0004), blockade of IL-6 alone did not delay tumor growth. Thus, our findings reveal that dual IL-6 and CTLA-4 blockade enhances anti-PDAC immune responses; however, the mechanism required further investigation.

### Combined IL-6 and CTLA-4 blockade increases T cells in pancreatic tumors.

In light of previous research investigating the influence of IL-6 (12) and CTLA-4 (13–15) on T cells, we hypothesized that combined blockade would increase T cell infiltration into pancreatic tumors. IHC staining of MT5 tumors from mice treated with this therapy indicated increased T cells within PDAC tumors compared with mice receiving isotype control or single-agent therapies (Figure 1, C and D). Image quantification revealed both single-agent blockade of IL-6 (*P* = 0.0038) or CTLA-4 (*P* = 0.0035) increased CD3^+^ T cell infiltration versus isotype control-treated mice (Figure 1, D and E). Mice given combined therapy had more T cells infiltrating tumors compared with mice treated with either single-agent blockade of IL-6 (*P* = 0.035), CTLA-4 (*P* = 0.038), or isotype controls (*P* = 0.0001), indicating a superior effect of IL-6 and CTLA-4 blockade on T cell infiltration in this tumor model (Figure 1D).

### Systemic shifts in cells with Th1 phenotypes occur following IL-6 and CTLA-4 blockade therapy.

The role of IL-6 in regulating T cell differentiation has been documented (16–18), while evidence suggests CTLA-4 blockade promotes the differentiation of naive CD4^+^ T cells into Th2 and T follicular (Tfh) subsets (13, 19, 20). Therefore, we evaluated the impact of this combination treatment on splenic T cells from mice bearing MT5 tumors as a surrogate of systemic changes. Mice receiving combined IL-6 and CTLA-4 blockade had increased CD4^+^ splenocytes as compared with those treated with anti–IL-6 alone (*P* = 0.0255) and isotype control (*P* = 0.0552) (Supplemental Figure 1A; supplemental material available online with this article; https://doi.org/10.1172/jci.insight.155006DS1). Anti–CTLA‑4 alone or in combination with IL-6 blockade increased splenic cells with a Th1 phenotype (CD4^+^CCR6^–^CXCR3^+^CCR4^–^) as compared with mice treated with isotype control (*P* < 0.0001 and *P* = 0.0004, respectively) or anti–IL-6 alone (*P* = 0.0026 and *P* = 0.0268, respectively) (Figure 1, E and F). No change in cells with Th2 (CD4^+^CCR6^–^CXCR3^–^CCR4^+^) or Th17 (CD4^+^RORγt^+^) phenotypes were evident (Figure 1, E and F, and Supplemental Figure 1B, respectively). Unexpectedly, the proportion of splenocytes expressing Treg markers (CD4^+^CD25^+^FoxP3^+^) was higher in the combination group versus both isotype control (*P* = 0.0027) and anti–IL-6 (*P* = 0.0007) in this model (Supplemental Figure 1D). Because IL‑6 also regulates expansion of myeloid-derived suppressor cells (MDSCs) in PDAC (21), phenotypic properties of these cells were assessed. We found no change in frequency of either monocytic (CD11b^+^Ly6G^–^Ly6C^+^) or granulocytic (CD11b^+^Ly6G^+^Ly6C^lo^) populations in splenocytes across groups in this s.c. tumor model (Supplemental Figure 1, F and G). These data indicate a dual role for combined IL‑6 and CTLA-4 blockade in driving T cells to infiltrate the tumor and shifting Th phenotypes.

### Dual IL-6 and CTLA-4 blockade is effective in mice with orthotopic PDAC tumors.

**Figure 2:**
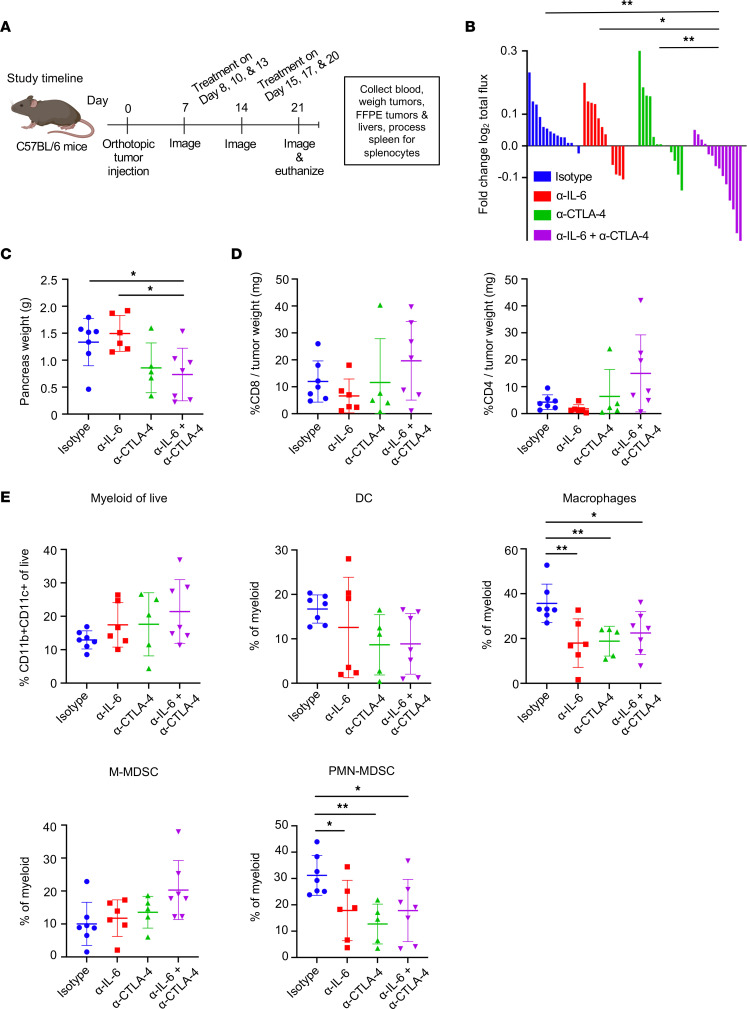
Combined blockade of IL-6 and CTLA-4 significantly inhibits tumor progression and modulates cell infiltration of tumors in an orthotopic murine model of pancreatic cancer. Study timeline in (**A**). C57BL/6 mice were orthotopically injected with 2 × 10^5^ KPC-luc cancer cells and treated 3 times a week for 2 weeks with Abs to IL-6 and CTLA-4 or isotype control Abs. (**B**) Tumor growth was tracked by BLI and the log_2_ fold change in total flux for each mouse was graphed as a bar. *P < 0.05; **P < 0.01. *P* values for significant comparisons using linear mixed models are as follows: isotype versus α–IL-6 + α–CTLA-4 (0.0002); α–IL-6 versus α–IL‑6 + α–CTLA-4 (0.017); and α–CTLA-4 versus α–IL-6 + α–CTLA-4 (0.009). Data are combined from 2 biologic replicates with similar results. Mouse numbers in each group were as follows: isotype, *n* = 15; α–IL-6**,** *n* = 12; α–CTLA-4, *n* = 13; and α–IL‑6 + α–CTLA-4, *n* = 14. (**C**) At the study endpoint, mice from 1 biologic replicate study were euthanized and the weight of each tumor was measured and graphed with symbols representing individual mice and mean displayed for each treatment group. *P* values for significant comparisons with ANOVA and LSD for pairwise comparisons are as follows: isotype versus α–IL-6 + α–CTLA-4 (*P* = 0.03) and α–IL-6 versus α–IL‑6 + α–CTLA-4 (0.008). (**D**) Flow cytometry was performed on TILs with Abs against CD8 and CD4 to assess T cells as a percentage of tumor weight. (**E**) TILs were also stained with a panel of Abs to delineate myeloid cells (CD11b^+^/CD11c^+^), DCs (I-A/E^+^ within CD11b^+^/CD11c^+^), macrophages (CD64^+^CD24^–^ within CD11b^+^/CD11c^+^), monocytic MDSCs (M‑MDSCs; Ly6C^+^Ly6G^–^ within CD11b^+^/CD11c^+^), and PMN-MDSCs (Ly6G^+^Ly6C^–^ within CD11b^+^/CD11c^+^). For **C**–**E**, mouse numbers were as follows: isotype, *n* = 7; α–IL-6**,** *n* = 6; α–CTLA-4, *n* = 5; and α–IL‑6 + α–CTLA-4, *n* = 7.

The efficacy of dual IL-6 and CTLA-4 blockade was next assessed in a more physiologically relevant murine model in which luciferase-expressing KPC cells (KPC-luc) are implanted into the pancreas of immune-competent mice, as detailed in Figure 2A. These cells express enhanced firefly luciferase that allows longitudinal biolumiscence imaging (BLI) of tumors. Longitudinal BLI indicated significant delay of tumor growth in mice receiving combined IL-6 and CTLA-4 blockade as compared with isotype control (p=0.002) or single agent (Figure 2B; *P* = 0.017 versus α–IL-6; *P* = 0.09 versus α–CTLA-4). Changes in total pancreas weight (containing tumors) at the study endpoint mirrored the efficacy of this treatment combination detected via BLI (Figure 2C) and indicated total pancreas weight from mice receiving dual IL-6 and CTLA-4 blockade were smaller than untreated animals given isotype control Abs (*P* = 0.03) or α–IL-6 alone (*P* = 0.008). All mice had primary tumors localized to the pancreas tail, while mice with advanced disease had tumor extensions into the peritoneum. Importantly, these data were reproducible, as similar efficacy data were obtained using a second murine model, whereby mice had panc02 tumors in the pancreas (Supplemental Figure 2). Upon study completion, tumors were harvested, and tumor-infiltrating lymphocytes (TILs) analyzed by flow cytometry to survey the immune features of the PDAC tumors. There was no significant difference in frequency of CD8^+^ T cells among groups, but a trend toward an increased frequency of CD4^+^ T cells in tumors from mice treated with dual IL-6 and CTLA-4 blockade, although this did not achieve statistical significance (*P* = 0.06) due to variability within the model (Figure 2D). Further evaluation of myeloid populations in this model indicated that PMN-MDSCs and macrophages were decreased in response to blocking IL-6, CTLA-4, or the combination as compared with mice receiving isotype control Abs (*P* < 0.05; Figure 2E). In contrast, no differences in monocytic MDSCs or DCs were evident in tumors between treatment groups (Figure 2E).

### Combined IL-6 and CTLA-4 blockade supports Th1 cytokines that crosstalk to facilitate chemokine production from tumor cells.

**Figure 3:**
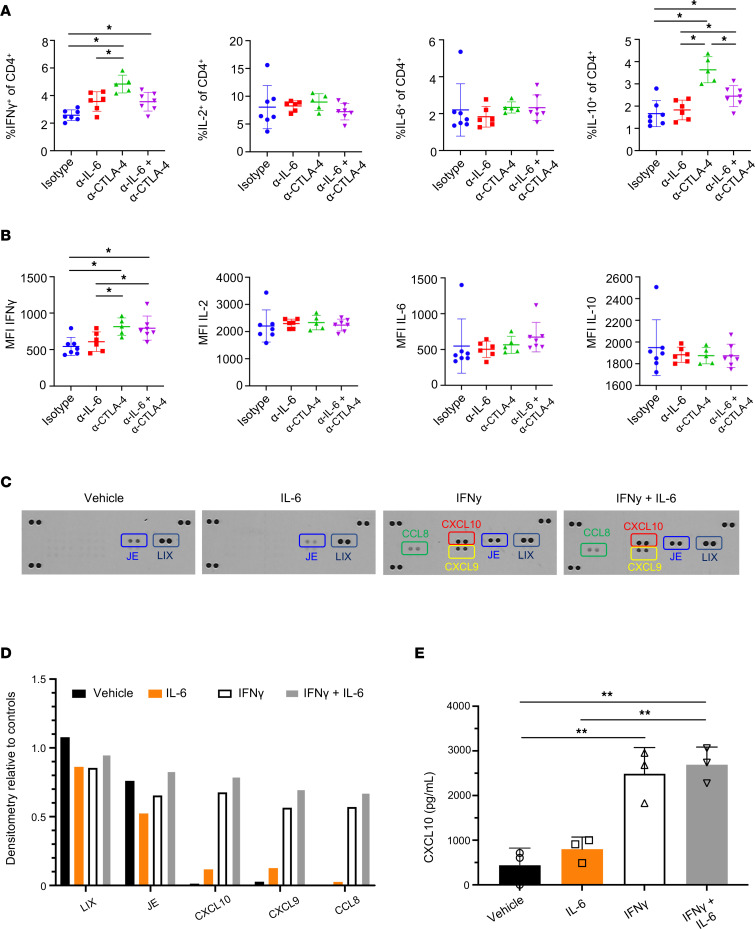
Combined blockade of IL-6 and CTLA-4 promotes IFN-γ production by CD4 T cells, which can elicit changes in chemokine production by pancreatic tumor cells. Splenocytes were isolated from mice bearing orthotopic KPC-luc following 2 weeks of Ab treatment as described in Figure 2. These cells were then stimulated with PMA/Ionomycin and assessed for intracellular cytokine production by flow cytometry. Mouse numbers in each group were as follows: isotype, *n* = 7; α–IL-6**,** *n* = 6; α–CTLA-4, *n* = 5; and α–IL‑6 + α–CTLA-4, *n* = 7. (**A**) The percentage of CD4^+^ T cells with intracellular cytokines (IFN-γ, IL-2, IL-6, and IL-10) were quantified by flow cytometry for each mouse with mean ± SD. *P < 0.05. (**B**) Graph shows the MFI of staining for each cytokine within the CD4^+^ T cells. (**C**) KPC-luc cells were plated at 2 × 10^5^ cells per well in 6 well plates and then stimulated with 10 ng/mL IFN-γ, 10 ng/mL IL-6, both, or vehicle control for 24 hours. Resulting supernatants were collected and analyzed using the Proteome Profiler Mouse Chemokine Array Kit as a screening tool. Shown are the resulting images of a chemokine membrane exposed to supernatants of KPC-luc cells from each treatment condition. (**D**) The relative densitometry to loading controls for LIX, JE, CXCL10, CXCL9, and CCL8 as detected by the chemokine array are graphed for each treatment condition. (**E**) Supernatants from replicates (*n* = 3) of the experiment described in **C** were quantified by ELISA and the resulting concentrations were graphed for CXCL10. Data are shown as the mean ± SD for each group. **P < 0.01 using ANOVA and LSD for pairwise comparisons.

As more Th1 cells were detected (based on chemokine signature phenotype) in mice following treatment with dual IL-6 and CTLA-4 blockade therapy (Figure 1F), we were prompted to consider the direct effects of our dual therapy on regulating the cytokine function in T cells. To address their capacity to secrete Th1-like cytokines, we stimulated splenic T cells from mice bearing orthotopic KPC-luc tumors in each of the 4 treatment groups with PMA/Ionomycin and assessed their intracellular cytokine production of IFN-γ, IL-2, IL-6, and IL-10 by flow cytometry. Both monotherapy CTLA‑4 blockade and dual IL-6/CTLA-4 blockade therapy mediated a predominant Th1 cytokine profile based on production of IFN-γ (Figure 3, A and B). Conversely, no changes in IL‑2 and IL-6 production were observed. Finally, we found that T cells from mice treated with anti–CTLA-4 Abs secreted IL-10 at high levels. To test if IFN-γ would be most induced with a dual blockade of IL-6 and CTLA-4 in an antigen-specific system, we next cultured these blocking Abs in the presence of transgenic CD4^+^ T cells, which express a T cell receptor (TCR) that recognizes a tyrosinase-related protein (TRP-1). Consistent with our findings in the MT5 model, TRP-1 CD4^+^ T cells treated with IL-6– and CTLA-4–blocking Abs secreted more IFN‑γ when restimulated with cognate antigen than untreated controls (Supplemental Figure 3A). This work suggests dual blockade fosters the generation of CD4^+^ T cells with a Th1 phenotype.

Previous research also suggests IL-6 alters classical IFN-γ response genes, including chemokines for T cell trafficking (22). To define how IL-6 and IFN-γ induce chemokines by PDAC cells, we surveyed chemokine production by murine MT5 or KPC-luc tumor cells following in vitro stimulation with IFN-γ and/or IL-6 cytokines. Analysis of supernatants by chemokine array indicated abundant production of lipopolysaccharide-induced CXC chemokine **(**LIX) and JE/Monocyte chemotactic protein 1 (JE/ MCP-1), regardless of treatment condition (Figure 3, C and D). Notably, upregulation of IFN-γ–induced canonical chemokines was evident, including CXCL10 and CXCL9, which ligate the CXCR3 receptor (23–26). CCL8 (MCP-2) was also upregulated by IFN-γ (Figure 3, C and D). The upregulation of CXCL10 was further validated by ELISA as a canonical CXCR3-binding chemokine (Figure 3E and Supplemental Figure 4, A and B). However, no change in chemokines was observed after treatment of MT5 or KPC-luc cells with IL-6 alone, nor did combined stimulation with IL-6 and IFN-γ differ significantly from IFN-γ alone as quantified by ELISA (Figure 3, C–E, and Supplemental Figure 4, A and B).

### CXCR3^+^ T cells infiltrate PDAC tumors in response to dual IL-6/CTLA-4 blockade.

**Figure 4:**
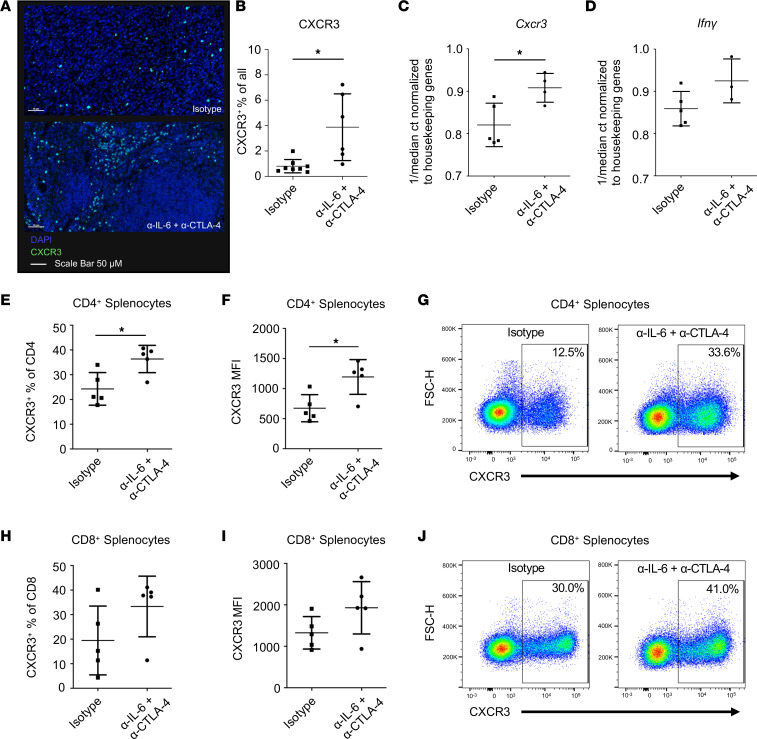
CXCR3^+^ T cells in PDAC tumors following dual IL-6/CTLA-4 blockade. C57BL/6 mice were orthotopically injected with 2 × 10^5^ KPC-luc cancer cells and treated 3 times a week for 2 weeks with Abs to IL-6 and CTLA-4 or isotype control Abs. (**A**) FFPE tumors (*n* = 6/group) were stained for DAPI (blue) and CXCR3 (green) by IHC and imaged using a Vectra Polaris immunofluorescent slide scanner (20×). Scale bars: 100 μM. (**B**) Whole slide scans were analyzed using Qupath and the percentage of all cells positive for CXCR3 is graphed. Each symbol represents a unique mouse. *P = 0.0371 with ANOVA and 2-tailed *t* test for pairwise comparisons. (**C**) CD3^+^CD45^+^ TILs were enriched from tumors of mice (*n* = 5/group) treated with Abs to IL-6 and CTLA-4 or isotype control Abs. RNA was isolated and analyzed by PCR for the expression of *Cxcr3* or (**D**) *Ifng*. Median Ct values were normalized to housekeeping genes and plotted as indicated. *P = 0.0223 with ANOVA and 2-tailed *t* test for pairwise comparisons. (**E**) Splenocytes from these mice were stained for CD3, CD4, CD8, and CXCR3. The percentage of CD4^+^ T cells expressing CXCR3 and (**F**) the MFI of CXCR3 on CD4^+^ T cells is graphed with (**G**) representative flow plots of CXCR3 staining shown. (**H**) The percentage of CD8^+^ T cells expressing CXCR3 and (**I**) the MFI of CXCR3 on CD8^+^ T cells is graphed with (**J**) representative flow plots of CXCR3 staining shown. * indicates significance with ANOVA and 2-tailed *t* test for pairwise comparisons. CXCR3^+^ is a percentage of CD4 (*P* = 0.0132). CXCR3 MFI on CD4^+^ T cells (*P* = 0.0430).

Based on our data in Figure 3, we hypothesized that Th1 cell trafficking into tumors via CXCR3 is a mechanism contributing to the efficacy of this therapy. To examine this question, we stained tumors for DAPI and CXCR3 and analyzed them by IHC (Figure 4A). Tumors from mice receiving combined IL-6 and CTLA-4 blockade had increased CXCR3^+^ T cells as a percentage of total cells compared with mice receiving isotype control Abs (Figure 4B). A subsequent study was conducted to validate whether TILs from mice expressed elevated CXCR3 in response to dual IL-6/CTLA-4 blockade. Here, CD3^+^ TILs were enriched by bead-based negative selection for CD3^+^ cells, followed by CD45^+^ selection to enrich CD3^+^CD45^+^ cells from established orthotopic tumors after 14 days of treatment with the combination or isotype control Abs. Endpoint tumor weight and changes in BLI are in Supplemental Figure 5, A and B. Since enzymatic digestion cleaves surface CXCR3, we evaluated RNA transcripts from TILs by PCR. These data revealed significantly greater CXCR3 on CD3^+^ TILs from mice treated with the combination therapy (Figure 4C). A trend toward increased IFN-γ transcripts was observed in these TILs, but this did not reach statistical significance (Figure 4D). Analysis of splenocytes from these same animals showed more circulating CD3^+^CD4^+^CXCR3^+^ T cells and a higher MFI for CXCR3 on these cells as well (Figure 4, E–G). In contrast, no significant difference in CXCR3 was observed on CD8^+^ T cells from each treatment group (Figure 4, H–J).

### CXCR3-dependent efficacy of combined IL-6 and CTLA-4 blockade.

**Figure 5:**
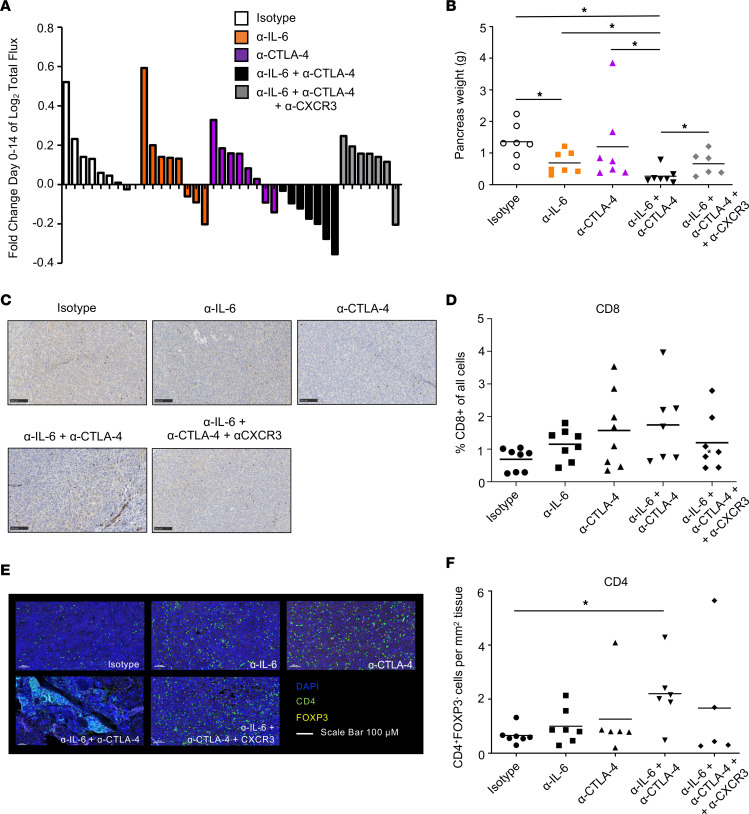
Dual IL-6/CTLA-4 blockade controls murine orthotopic pancreatic tumors in a CXCR3-dependent manner and modulates intratumoral T cells. (**A**) Mice bearing orthotopic KPC-luc tumors were treated with isotype control Abs, Abs to IL-6, CTLA-4, both Abs combined, or dual IL-6 and CTLA-4 blockade together with Abs to CXCR3. Mouse numbers were as follows: isotype, *n* = 8; α–IL-6**,** *n* = 8; α–CTLA-4, *n* = 8; α–IL‑6 + α–CTLA-4, *n* = 7; and α–IL‑6 + α–CTLA-4 + α-CXCR3, *n* = 7. Tumor growth was tracked by BLI and log_2_ fold change in total flux for each mouse was graphed as a bar. Using linear mixed models, all groups were determined to be significantly different to mice receiving dual IL-6 and CTLA-4 blockade (*P* < 0.05). *P* values for significant comparisons are as follows: isotype versus α–IL-6 + α–CTLA-4 (0.0003); α–IL-6 versus α–IL-6 + α–CTLA-4 (0.0104); α–CTLA-4 versus α–IL-6 + α–CTLA-4 (0.0039); and α–IL-6 + α–CTLA-4 versus α–IL-6 + α–CTLA-4 + α-CXCR3 (0.0029). (**B**) The total weight (grams) of each tumor-containing pancreas was collected and graphed as the mean for each group. *P < 0.05. *P* values for significant comparisons using ANOVA and LSD for pairwise comparisons are as follows: isotype versus α–IL-6 + α–CTLA-4 (0.0005); α–IL-6 versus α–IL-6 + α–CTLA-4 (0.0224); isotype versus IL-6 + α–CTLA-4 + α-CXCR3 (0.0247); and α–IL-6 + α–CTLA-4 versus α–IL-6 + α–CTLA-4 + α-CXCR3 (0.0405). FFPE tissue slices of tumors from mice in (**A**) were stained for CD8 by IHC. (**C**) Representative images of IHC staining (20×) for CD8 in tumors. Scale bars: 100 μM. (**D**) Infiltrating CD8 T cells were quantified using Qupath and graphed as % CD8 of all cells. Results are displayed as the mean for each group. (**E**) FFPE tissue slices from tumors were stained for CD4 and FOXP3 with DAPI counterstain. Representative images are displayed with CD4 staining (green), FOXP3 (red), and DAPI (blue). Scale bars: 100μM. (**F**) After scanning slides using a Perkin Elmer Vectra Polaris fluorescent slide scanner, the percentage of CD4^+^FOXP3^–^ cells were quantified with Qupath and graphed as the mean for each treatment group. *P = 0.0297 between isotype and receiving dual IL‑6/CTLA-4 blockade using ANOVA and LSD for pairwise comparisons.

To determine if CXCR3 receptor interactions were essential for the T cell response, we Ab blocked CXCR3 in mice receiving combination therapy. Mice receiving combined IL-6 and CTLA-4 blockade experienced consistent tumor regression by BLI (Figure 5 and Supplemental Figure 5A). As expected, we found that concurrent CXCR3 blockade significantly inhibited the efficacy of combination therapy, leading to results similar to treatment with isotype controls (Figure 5, A and B). No profound change in tumor growth by BLI was evident between mice treated with isotype controls or single-agent therapy. Post-mortem pancreas weight at study endpoint confirmed growth inhibitory effects of the combination were significant, as compared with treatment with anti–CTLA-4 or isotype controls (*P* = 0.0778 and *P* = 0.0005, respectively; Figure 5B). Similar to prior studies (Figure 2, B and C), the primary tumor localized to the pancreas tail was confirmed post mortem, and mice with advanced disease had small peritoneal extensions. In this study, 4 mice in the combination therapy group had no tumors, but pancreatic weight was recorded. Notably, this CXCR3-dependent mechanism of concurrent IL-6 and CTLA‑4 blockade was confirmed in mice bearing s.c. MT5 tumors (Supplemental Figure 5C).

### Dual blockade therapy and infiltration of T cells into PDAC tumors.

Changes in the tumor microenvironment that may explain efficacy of this combination were surveyed by IHC. Although prior observations show IL-6 is derived from fibroblasts in PDAC (21, 27), α–smooth muscle actin (α-SMA) staining was not significantly different between groups (Supplemental Figure 6, A and B). A trend toward increased CD8^+^ T cells in tumors from mice receiving dual blockade was seen, but was not significant (Figure 5, C and D). Although no differences in CD4^+^FoxP3^+^ T cells emerged (Supplemental Figure 6C), a consistent increase in CD4^+^ T cells lacking FOXP3 expression was present in tumors from mice receiving combined IL‑6 and CTLA‑4 blockade compared with controls (*P* = 0.0297) or to mice receiving anti–IL-6 alone (*P* = 0.0439) (Figure 5, E and F). There was a trend toward reduced CD4^+^ T cells in tumors from mice treated with CXCR3-directed Abs and the combination of IL-6 and CTLA-4 blockade, although it did not reach statistical significance.

### Dual blockade therapy expands systemic TBET^+^ and GATA3^+^CD4^+^ T cells in an orthotopic PDAC model.

**Figure 6:**
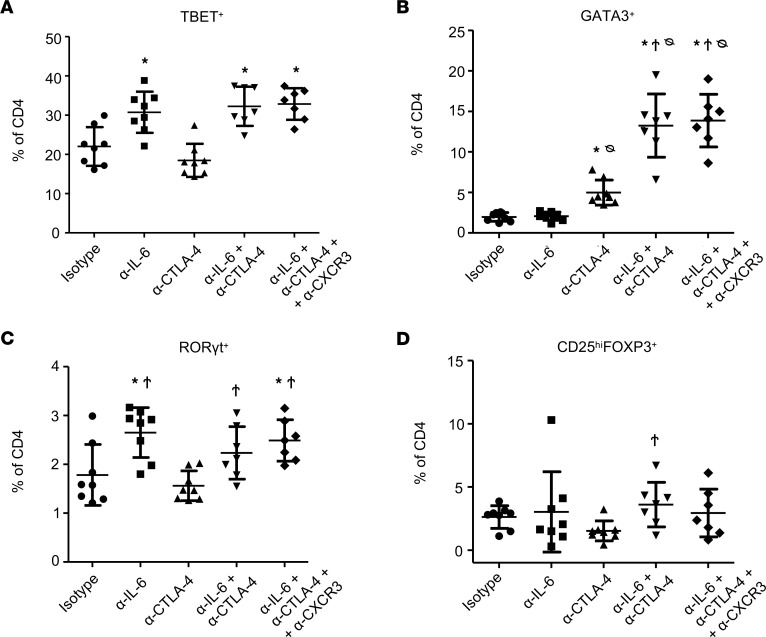
Combined blockade of IL-6 and CTLA-4 in mice bearing orthotopic pancreatic tumors results in splenic changes in CD4 Th cells. Splenocytes were isolated from the mice receiving treatment as stated in Figure 4. Cells were stained for CD3, CD4, and CD8 surface markers with Ghost 780 viability stain to mark dead cells. After fixation and permeabilization, cells were stained for the transcription factors TBET, GATA3, RORyt, and FOXP3. The percentage of CD4^+^ T cells that were (**A**) TBET^+^, (**B**) GATA3^+^, (**C**) RORγt^+^, or (**D**) CD25^hi^FOXP3^+^ were graphed as the mean ± SD with symbols indicating significance (*P* < 0.05) compared with *isotype control mice, ^Ϯ^α-CTLA-4–treated mice, or ^ᴓ^α-IL-6–treated mice. *P* values for significant comparisons using ANOVA and LSD for pairwise comparisons are listed in Supplemental Table 2.

We next investigated the effects of treatment on systemic T cell phenotypes in the orthotopic model. Flow cytometry (Figure 6A) recapitulated observations of Th1 immunity, consistent with splenocyte data in the s.c. model (Figure 1, E and F). Robust expansion of CD3^+^CD4^+^TBET^+^ (Th1) T cells was observed in splenocytes from mice receiving combined IL-6 and CTLA-4 blockade (*P* = 0.0015) or anti–IL-6 alone (*P* = 0.0041) versus isotype-treated mice (Figure 6A). We also observed significantly higher splenic CD3^+^CD4^+^GATA3^+^ (Th2) T cells in mice treated with the combination compared with isotype controls (*P* = 0.0003; Figure 6B). Of note, CD3^+^CD4^+^GATA3^+^ T cells were more abundant in mice receiving the combination blockade versus those treated with anti–CTLA-4 alone (*P* =.0012; Figure 6B). Thus, a unique increase in both TBET^+^ and GATA3^+^CD4^+^ T cells was observed in mice treated with combined IL-6 and CTLA-4 blockade. We also observed no difference in frequency of CD3^+^CD4^+^RORγt^+^ cells or in Tregs, defined phenotypically as CD3^+^CD4^+^CD25^hi^FOXP3^+^, from mice receiving combined IL-6 and CTLA-4 blockade as compared with isotype control-treated mice (Figure 6, C and D). There were no differences in these systemic biomarkers between mice receiving only the combination or the combination together with CXCR3-targeted Abs (Figure 6, A–D).

We observed a higher percentage of CD4^+^ T cells positive for PD-1 in splenocytes from mice treated with anti–IL-6 alone or combined with CTLA-4 blockade compared with isotype control or single-agent anti–CTLA-4 (Supplemental Figure 7A). Contrasting data for CD4^+^ T cell subsets, few changes were observed in composition of splenic CD8^+^ T cells of mice treated with single-agent or combination therapy. However, mice receiving combined IL-6 and CTLA-4 blockade demonstrated significantly more CD8^+^PD-1^+^ T cells than mice receiving isotype control or anti–IL‑6 Abs (Supplemental Figure 7B). Immunologically, this strategy impacted CD4^+^ T cells, driving increases in both Th1 and Th2 immunity, with more limited systemic changes in CD8^+^ T cells.

### Combined IL-6 and CTLA-4 blockade is dependent upon CD4^+^ and CD8^+^ T cells.

**Figure 7:**
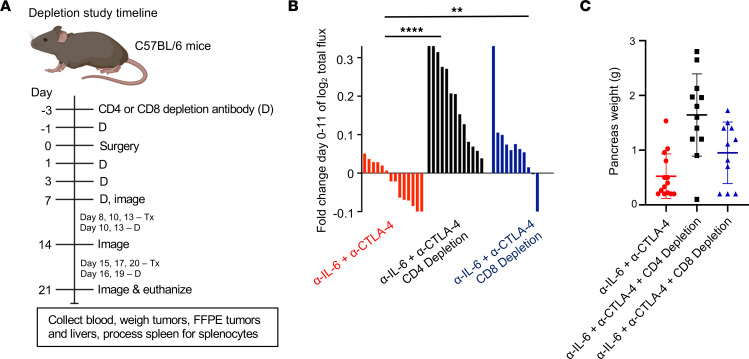
Both CD4^+^ and CD8^+^ T cells are required for antitumor responses to orthotopic pancreatic tumors in mice treated with combined IL-6 and CTLA-4 blockade. Female C57BL/6 mice, 6–8 weeks of age, were orthotopically injected with 2 × 10^5^ KPC-luc cancer cells and imaged 1 week later by BLI to confirm tumor establishment. Mouse numbers in each group were as follows: α–IL‑6^+^α–CTLA-4, *n* = 14; α–IL‑6^+^α–CTLA-4^+^CD4 depletion, *n* = 13; and α–IL‑6^+^α–CTLA-4^+^CD8 depletion, *n* = 11. (**A**) Study timeline for the administration of CD4- or CD8-depleting Abs relative to orthotopic injection and subsequent administration of IL-6– and CTLA-4–blocking Abs or isotype control Abs. (**B**) Tumor growth for each mouse was measured over time by BLI and the fold change in log_2_ of total flux for each mouse was graphed as a bar. BLI data for controls in this experiment are included in Supplemental Figure 8. Significance in BLI was determined using linear mixed models, **P = 0.0087, ****P < 0.0001. (**C**) At the study endpoint, mice were euthanized and the weight (grams) of each pancreas containing tumor was measured and graphed with symbols representing individual mice and the mean displayed for each treatment group, significance was determined using ANOVA and LSD for pairwise comparisons. Mice depleted of CD4 cells had significantly higher pancreas weight compared with mice receiving combination therapy without depleting antibodies.

As T cells were elevated in tumors from animals given our dual therapy, we next posited that the efficacy of this combination therapy was dependent on either CD4^+^ or CD8^+^ T cells. For these studies, we again used the more physiologically relevant model in which KPC-luc cancer cells were orthotopically implanted into the pancreas of immune-competent mice. CD4^+^ or CD8^+^ T cells were Ab depleted in mice bearing orthotopic KPC-luc tumors prior to treatment with combined IL-6 and CTLA-4 blockade (Figure 7A). We confirmed CD4 or CD8 depletion by flow cytometric analysis of cells from the spleens and in tumors at endpoint (Supplemental Figure 8, A–C). Longitudinal BLI data indicated CD4^+^ T cell–dependent efficacy of combined IL-6 and CTLA-4 blockade, as CD4^+^ T cell–depleted mice receiving therapeutic Abs had accelerated tumor growth progression compared with mice receiving only the combination therapy (Figure 7B). Tumor progression in some animals was striking and faster than in mice receiving isotype control Abs. CD8^+^ T cell depletion also impacted tumor growth, albeit not to the magnitude of CD4^+^ T cell depletion (Figure 7B and Supplemental Figure 8B). To complement trends from BLI data, total pancreas and tumor weights were measured post mortem (Figure 7C). One mouse receiving dual IL-6/CTLA-4 had no evidence of tumor, but the pancreas weight was recorded. These data confirmed efficacy in multiple murine PDAC models and highlighted the requirement for both CD4^+^ and CD8^+^ T cells.

## Discussion

IL-6 is elevated in patients with pancreatic cancer, suggesting this cytokine plays a role in blunting T cell immunity (28). However, Abs that neutralize IL-6 have not been effective as single agents in patients with PDAC tumors (29). Moreover, blockade of CTLA-4 has limited efficacy as a single agent in this aggressive disease (30, 31). Here, we demonstrate that combined blockade of these targets elicits potent antitumor activity in 3 different pancreatic tumor models: s.c. MT5, orthotopic KPC-luc, and orthotopic Panc02 pancreatic tumors. The efficacy of concurrently targeting IL-6 and immune checkpoints was, to the best of our knowledge, first established by our group (27). In these studies, blockade of IL-6 and PD-L1 in murine PDAC models significantly inhibited tumor growth while promoting effector CD8^+^ T cell infiltration of tumors (27). This effect has since been reproduced in murine models, including glioblastoma, colorectal cancer, and melanoma (32–34). IL-6 blockade may also have an advantage through limiting autoimmune toxicity and retaining efficacy from immune checkpoint blockade (35). The data from this report are important in lending flexibility to clinical translation, whereby multiple immune checkpoint Abs may have efficacy via nonoverlapping mechanisms. The current study highlights potentially novel immunomodulatory mechanisms observed upon neutralizing IL-6 alongside CTLA-4 blockade that are distinct from PD-1/PD-L1 blockade.

Results from this study indicate what we believe to be a unique mechanism of action when compared with combined IL-6 and PD-1/PD-L1 blockade. Previously, the efficacy of dual IL-6 and PD-1/PD-L1 blockade was dependent on CD8^+^ but not CD4^+^ T cells. While CD8^+^ T cell depletion significantly restored tumor growth in mice receiving dual IL-6/CTLA-4 blockade, CD4^+^ depletion resulted in pronounced tumor growth. Furthermore, in vivo CXCR3 blockade revealed that the efficacy of anti–IL‑6/CTLA-4 combination therapy was reliant on this chemokine-receptor interaction. Additionally, combined IL-6 and PD-1/PD-L1 blockade reduced α-SMA^+^ stromal content, while these changes were not observed following CTLA-4 and IL-6 blockade (27). This discrepancy may be due to differential effects on IL-6 producing fibroblast subsets from therapy (36, 37). Of note, these conclusions were derived from data in mice with orthotopic, transplantable tumors, rather than spontaneously arising genetically engineered mouse models, which might approximate different stromal features. We also acknowledge that these orthotopic models, while a powerful tool, do indeed harbor some variability as some measures of tumor burden are taken as a function of the weight of the total pancreas. This highlights the value of interpreting data in the context of both pancreas weight and BLI when drawing conclusions related to efficacy and immune biomarkers.

Previous studies investigating T cell responses to PDAC propose differing results with respect to the effect of CTLA-4 on CD4^+^ or CD8^+^ T cells (15, 38). Here we observed a dependence on both CD4^+^ and CD8^+^ T cells to mediate the antitumor effects of combined IL-6 and CTLA-4 blockade. CD4^+^ Th support of CD8 cells may provide heterogeneous T cell responses that mediate PDAC regression. Recent evidence describes the need for CD4^+^ and CD8^+^ T cell activation in antitumor responses (39). This study reinforces the importance of CD4^+^ T cells in optimal efficacy of immunotherapy.

We hypothesized that the efficacy of IL-6 and CTLA-4 blockade may be mediated in part by tumor-derived chemokines that enhance lymphocyte trafficking into tumors. Studies of anti–CTLA-4 alone in murine PDAC models report the presence of tumoral CD4^+^ T cell infiltration from lymph nodes (15). Our in vitro studies demonstrate combined IL-6 and CTLA-4 blockade elicits increases in IFN-γ–producing CD4^+^ T cells in the context of antigen-specific activation (13, 14). While we observed this effect may be driven by IL-6 blockade, previous studies in prostate cancer observed elevated numbers of IFN-γ–producing CD4^+^ T cells upon administration of CTLA-4–blocking Abs. IFN-γ is appreciated as a multifaceted soluble factor capable of directly inhibiting tumor cell growth, driving immune activation, and stimulating production of IFN response genes by tumor cells (40–43). One group has demonstrated the activation of IFN response genes in cancer cells occurs, suggesting this may contribute to the efficacy of CTLA-4 blockade, such that CTLA-4 blockade fails in patients with defects in these genes (44). While the MT5 and KPC-luc lines used in this study secrete the CXCR3 ligands CXCL9 and CXCL10, it is uncertain if patients with defects in these pathways would benefit from dual blockade of IL-6 and CTLA-4. Indeed, recent reports support the importance of the CXCR3 chemokine axis for mediating responses to ICI (45), strengthening our observations here. Certainly other chemokine/receptor interactions can also contribute to immunotherapy response in pancreatic cancer, as evident from recent CCR2 and CCR5 inhibitors, which may be of interest for future study (46). We also acknowledge that our study captures immune infiltration data that are taken at a single time point and, thus, may not represent all phases of response to this therapy. Overall, our in vivo studies depleting CD4^+^ T cells and employing the CXCR3-blocking Ab highlight infiltration of CXCR3-expressing T cells as a potential mechanism that contributes to tumor regression in the context of this combination therapy.

While this therapy elicits antitumor activity, ICI has the potential for toxicity in patients. Attempts to ameliorate the autoimmune toxicities of ICI including anti–CTLA-4 and anti–PD-1/PD-L1–targeted Abs revealed the IL-6R blocking Ab tocilizumab is effective in patients refractory to steroids (35, 47, 48). A recent report demonstrated tocilizumab alongside pembrolizumab in melanoma prevented exacerbation of Crohn’s disease and allowed durable antitumor immune responses (47). Similarly, a case report showed tocilizumab in a patient with pulmonary adenocarcinoma completely resolved immune-related toxicities to nivolumab including oropharyngeal mucositis and esophagitis (49). Other data in preclinical models coupling melanoma with experimental autoimmune encephalitis demonstrate that IL-6 blockade can improve antitumor efficacy without exacerbating autoimmune toxicity. In this same report, a retrospective analysis of 31 patients with melanoma further showed concurrent medication with IL-6–blocking agents (for other indications) could mitigate immune-related adverse events without compromising the antitumor activity of immune checkpoint blockade (35). Finally, use of IL‑6R–blocking Abs with chimeric antigen receptor T cells (CARTs) produced encouraging results while enhancing patient safety (48). This emerging use of IL-6/IL-6R blockade to limit ICI-associated toxicities has led to clinical trials exploring the use of IL-6 blockade for improved safety of these therapies (NCT03601611).

Despite these encouraging results, efforts to apply IL-6 or IL-6R blockade prospectively with therapeutic intent in clinical trials have lagged behind preclinical data, possibly due to resistance to repurpose these drugs from autoimmunity into the oncology setting. To date, only a limited number of clinical trials (NCT04191421, NCT04258150, and NCT03193190) are combining IL-6 blockade therapy with ICI in PDAC. Continued experience with IL-6 and ICI combinations across solid tumors will inform the field regarding efficacy and the ability to limit autoimmune sequelae.

## Methods

### Cell lines and Abs.

Murine MT5 (Kras^LSL-G12D^, Trp53^LSL-R270H^, and Pdx1-cre) pancreatic cells were from David Tuveson (Cold Spring Harbor Laboratory) and were cultured in RPMI with 10% FBS and Antibiotic:Antimycotic Solution (GeminiBio). Murine KPC-luc (Kras^LSL-R270H^, p53^–/–^, and Pdx1-cre) cells were from Craig Logsdon (MD Anderson Cancer Center). Panc02 was provided by Shari Pilon-Thomas (H. Lee Moffitt Cancer Center, Tampa, Florida, USA). KPC-luc and Panc02 cells were cultured in DMEM with 10% FBS and Antibiotic:Antimycotic Solution. All cell lines were confirmed to be mycoplasma free by both internal and external mycoplasma testing. Murine Abs to IL-6 (clone MP5-20F3), CTLA-4 (clone 9D9), CXCR3 (clone CXCR3-173), or isotype controls (clones LTF-2 for s.c. or HRPN for orthotopic studies, MCP-11, and polyclonal Armenian hamster IgG, respectively) were from BioXcell. Anti-mouse CD8a- (clone 2.43) and anti-mouse CD4-depleting Abs (clone GK1.5) were from BioXcell.

### In vivo efficacy studies.

For initial in vivo efficacy studies, 1 x 10^6^ MT5 tumor cells were injected s.c. in the flank of female C57BL/6 mice (strain 000664, The Jackson Laboratory). Once tumors reached 50–100 mm^3^ (typically 7–10 days), Ab treatment was initiated. S.c. studies ended once tumors reached volumes meeting IACUC-mandated early removal. For orthotopic studies, 6- to 8-week-old female C57BL/6 mice underwent inhalation anesthesia with isoflurane. The abdomen was prepared, draped, and a midline laparotomy was made in the upper abdomen. Fascia was incised and spleen mobilized from the left upper quadrant and elevated to identify the pancreas tail. A 27.5 gauge needle was used to inject 2 x 10^5^ KPC-luc cells into the anterior medial body of the pancreas. Hemostasis was ensured, the spleen was returned to normal anatomic location, and fascia closed with running absorbable suture. Mice were awakened and monitored for 1 hour. Tumors grew for 7 days prior to randomization and confirmed by BLI using the IVIS Spectrum In Vivo Imaging system and luciferase. Mice were then randomized to treatment groups based on BLI. For all studies, Abs to IL-6, CTLA-4, or CXCR3 were delivered by i.p. injection thrice weekly every 2–3 days with a maximum number of 6 doses per mouse delivered throughout the course of treatment. For depletion studies, CD4- or CD8-depleting Abs were delivered at day –3, –1, +1, +3, and +7 relative to tumor implantation. Following confirmation of tumor establishment by BLI, depleting Abs were delivered twice weekly every 3–4 days until study endpoint.

### Abs for flow cytometry and IHC staining.

Abs, with clone names, used for flow cytometry and IHC are in Supplemental Table 1.

### Flow cytometry.

At completion of s.c. efficacy studies, tissues were harvested for immunophenotypic analyses of splenocytes and single-cell suspensions from tumors were assessed by flow cytometry as described (27). Analysis was performed on a LSRII flow cytometer (BD Biosciences) or a Cytek Aurora (Cytek). Splenocytes from mice bearing orthotopic tumors were stained with Ghost 780 dye to detect live cells and Abs for CD4 and CD8 T-cell phenotypes. Intracellular staining was performed using the eBioscience Foxp3/Transcription Factor Staining Buffer Set per manufacturer’s protocol.

### IHC analysis.

FFPE tumors from s.c. experiments underwent IHC analysis following staining with Ab against CD3 (catalog A0452, Dako). PerkinElmer’s Vectra multispectral slide analysis system was used to capture 40× magnification of images of tumors (10 images/tumor). inForm software quantified CD3-positive cells (Fast Red chromogen) within each image. Additional slices were stained for CD8 and α-SMA and scanned with an Olympus Nanozoomer whole slide scanner and analyzed using Qupath (CD8) or FIJI (NIH) for α-SMA. Orthotopic tumors were also FFPE. Dual stains for DAPI (Perkin Elmer) with CD4 and FOXP3 were performed using a Roche autostainer and detected with Opal 520 and Opal 630-conjugated secondary (Perkin Elmer), respectively. Slides were imaged using the Vectra Multispectral Imaging System version 2 (Perkin Elmer). Filter cubes for imaging were DAPI (440–680 nm), FITC (520–680 nm), Cy3 (570–690 nm), Texas Red (580–700 nm), and Cy5 (670–720 nm). Multispectral images were analyzed with Qupath (50).

### TRP-1 transgenic CD4^+^ T cell activation.

CD4^+^ TRP-1 transgenic T cells (51) were isolated from the spleen of RAG1^–^B^W^ TRP-1 TCR mice (strain 008684, The Jackson Laboratory), activated with TRP-1106-130 peptide (SGHNCGTCRPGWRGAACNQKILTVR) loaded at 1 μM concentration onto irradiated B6 splenocytes (10 Gy) at a 2:1 TRP-1/feeder cell ratio. TRP-1 cells were cultured with monoclonal Abs targeting CTLA-4 (10 μg/mL, clone 9D9), IL-6 (10 μg/mL, clone MP5-20F3), or isotype controls (10 μg/mL, IgG2b, or HRPN) with IL-2 (100 IU/mL). Cells were assessed 3 days after activation for cytokine production following PMA/Ionomycin stimulation. Briefly, cells were activated in PMA (30 nM) and Ionomycin (20 nM) (MilliporeSigma) with Monensin (2 μM) and Brefeldin A (5 μg/mL) (BioLegend) for 4 hours, followed by fixation and permeabilization for cytokine staining per protocol (BioLegend).

### In vitro evaluation of chemokine production.

KPC-luc or MT5 cells were plated at 2 x 10^5^ cells per well in 6 well plates. Media was supplemented with 10 ng/mL IL-6 (PeproTech), 10 ng/mL IFN-γ (PeproTech), both cytokines combined, or vehicle for 24 hours. Supernatant was collected and spun at 1,000*g* then transferred to new tubes to limit cellular contamination. Supernatants were analyzed using Proteome Profiler Mouse Chemokine Array Kits (catalog ARY020, R&D Systems). Results were confirmed using a DuoSet ELISA kit (R&D Systems) for CXCL10.

### Statistics.

Data from s.c. studies obtained by flow cytometry, IHC, and tumor volumes were log transformed prior to analysis to meet model assumptions of normality and homoscedasticity. Tumor volume was modeled over time using mixed-effects regression with fixed effects for group, time, and the interaction between them. Random intercepts and slopes by mouse were included with an unstructured covariance matrix for random effects. Other outcomes were compared using 1-way ANOVA. *P* values less than 0.05 were significant.

For numeric covariates, the mean and standard deviation were calculated and presented. For IHC and splenocyte data, 1-way ANOVA was performed with univariate analysis. Least significant difference method (LSD) was used for pairwise multiple comparisons. Natural log transformation of BLI data was performed to achieve approximately normal distribution of data. For log-transformed data, linear mixed models were tested for significant change over time of each outcome and to detect significant difference of each outcome among treatments. Significance was set at 0.05. For in vitro data, natural log transformation was performed to normally distribute data. We then performed 1-way ANOVA and LSD to detect whether means significantly differed among treatment groups. All analyses were conducted in SAS v9.4 (SAS Institute). For experiments where statistical significance was detected, 2-tailed *t* tests were then utilized to determine pairwise significance.

### Study approval.

Animal studies were conducted under IACUC approval at The Ohio State University or Emory University. This study did not receive nor require ethics approval, as it does not involve human participants.

## Author contributions

MBW, CM, TAM, CMP, and GBL conceptualized and designed experiments. MBW, CM, and MYZ, carried out the animal studies. MBW performed the majority of the in vitro and post-in vivo analysis (flow cytometry, IHC). MP, HMK, BR, CJH, MRF, ANR, and JSB contributed to in vivo and in vitro experiments throughout the course of the study. ABF, RS, WEC, and BER provided guidance and consultation on study design and manuscript formation. ZC, GY, and CZ performed statistical analysis and provided guidance on powering and designing experiments. MBW, CM, MYZ, and GBL drafted the manuscript. All authors contributed to writing and drafting of the final manuscript.

## Supplementary Material

Supplemental data

## Version 1 03/07/2023

In-Press Preview

## Version 2 04/24/2023

Electronic publication

## Version 3 02/06/2025

The color legend in Figure 1F was swapped. This version fixes the error
