# Supplementary material for: Dual IL-6 and CTLA-4 blockade regresses pancreatic tumors in a T cell– and CXCR3-dependent manner
Source: JCI Insight. 2023 Mar 7;8(8):e155006. doi: 10.1172/jci.insight.155006 (PMC10243806; doi:10.1172/jci.insight.155006)
Supplement: Supplemental data [file jciinsight-8-155006-s008.pdf]

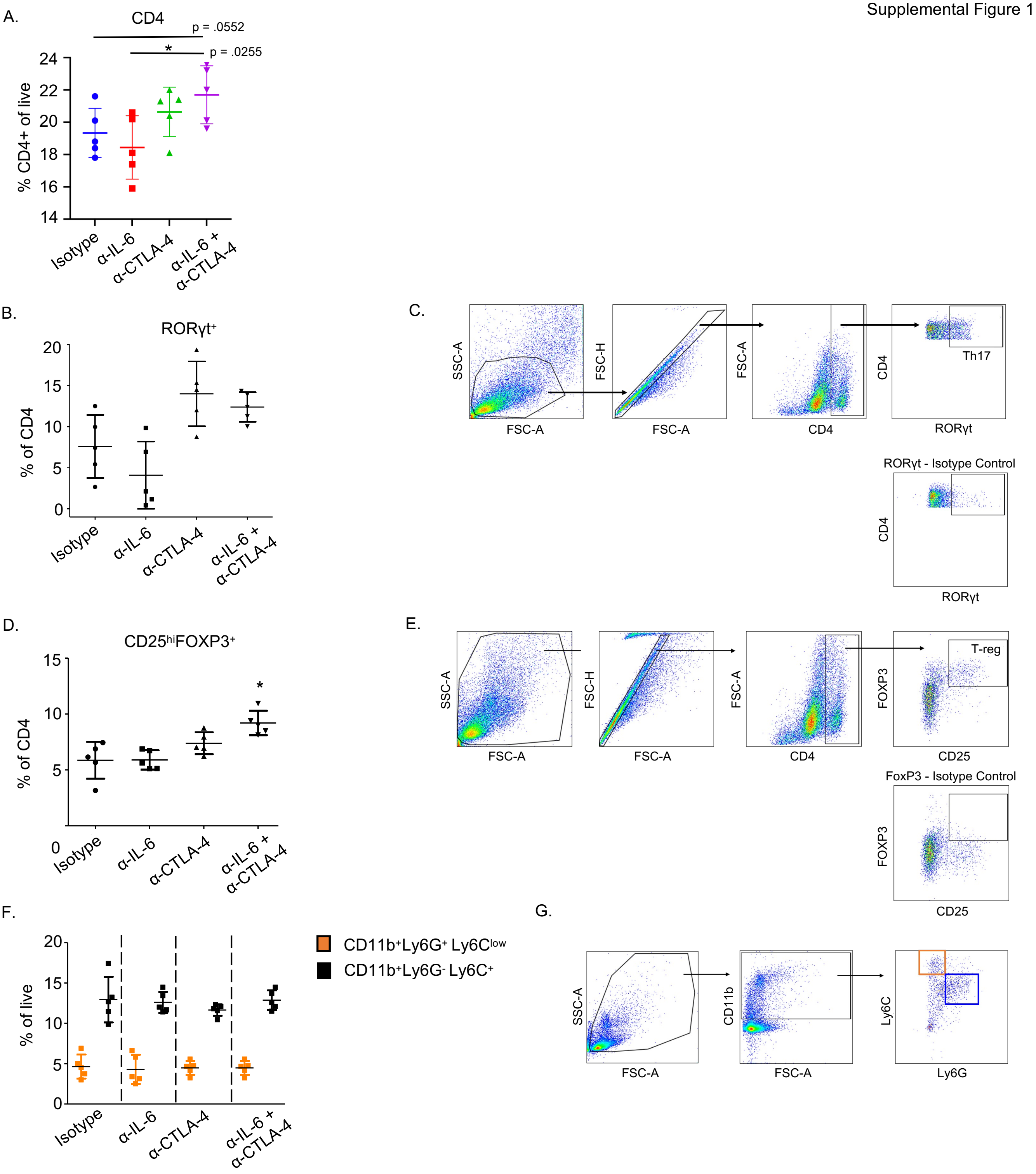

Supplemental Figure 1. Splenocytes were isolated from the spleens of mice from the subcutaneous therapeutic study described in **Figure 1. (A)** Splenocytes were stained with CD4 antibody and analyzed by flow cytometry. The percentage of $CD4^+$ cells of the live cell population were graphed as mean ± SD. **(B)** Splenocytes were stained with antibodies to CD4 and RORγt and analyzed by flow cytometry. The percentage of $CD4^+$ cells that were $ROR\gamma t^+$ were graphed as mean ± SD. **(C)** Gating strategy for the identification of Th17 cells ($CD4^+ROR\gamma t^+$) **(D)** Splenocytes were stained with antibodies to CD4, CD25 and FOXP3 and analyzed by flow cytometry. The percentage of $CD4^+$ cells that were $CD25^{hi}FOXP3^+$ were graphed as mean ± SD. * indicates significance ($P<.05$) compared to isotype control treated animals. **(E)** Gating strategy for the identification of Tregs ($CD4^+CD25^{hi}FOXP3^+$) **(F)** Splenocytes were stained with antibodies to CD11b, Ly6C and Ly6G as well and analyzed by flow cytometry. The percentage of live cells that were $CD11b^+Ly6G^+Ly6C^{low}$ or $CD11b^+Ly6G^-Ly6C^+$ were graphed as mean ± SD. **(G)** Gating strategy for the identification of monocytic-MDSCs ($CD11b^+Ly6G^-Ly6C^+$) and polymorphonuclear-MDSCs ($CD11b^+Ly6G^+Ly6C^{low}$).

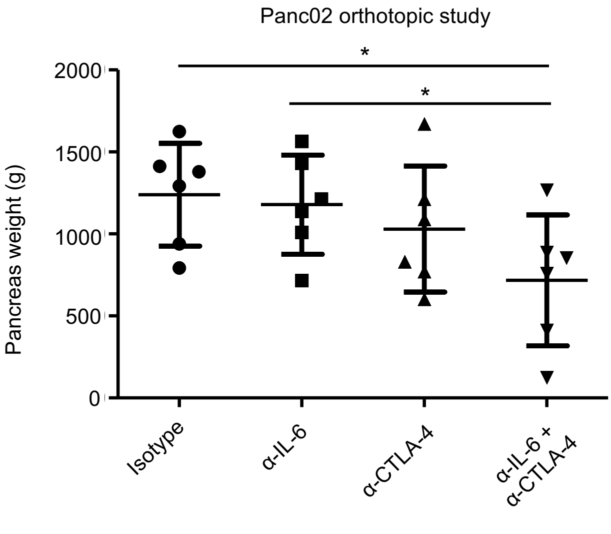

Supplemental Figure 2. 6-8wk old female C57BL/6 mice were orthotopically injected with $2e^5$ Panc02 cells and treated with blocking antibodies to IL-6, CTLA-4, the combination or Isotype controls. At study end tumors were resected and weighed. Average pancreas weight (grams) withing each group is shown. Weights were analyzed by one-way ANOVA to determine if significantly different followed by unpaired students t-test for pairwise comparisons.

A.

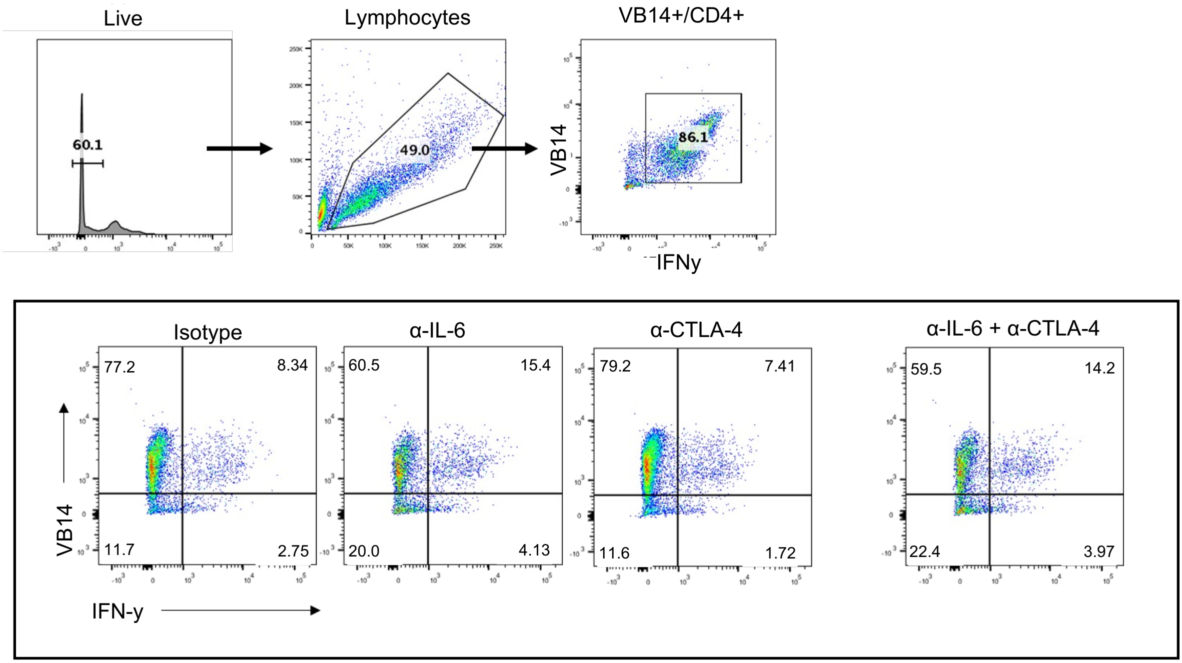

B.

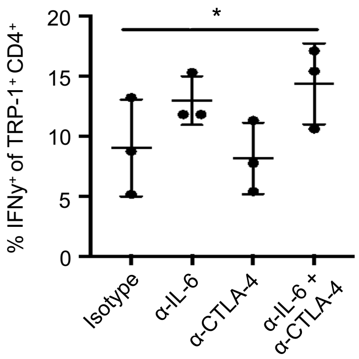

Supplemental Figure 3. (A) Trp-1 specific $CD4^+$ T cells were stimulated in the presence of antibodies to IL-6, CTLA-4, the combination of both or isotype control antibodies. The percentage of cells expressing Vβ14 and IFN-γ were quantified by flow cytometry. **(B)** Graph shows percentages of $CD4^+$ T cells expressing IFN-γ for each mouse with mean ±SD. $CD4^+$ T cells expressed more IFN-γ in the presence of dual CTLA-4 and IL-6 blockade compared to isotype control antibodies ($p=0.0217$)

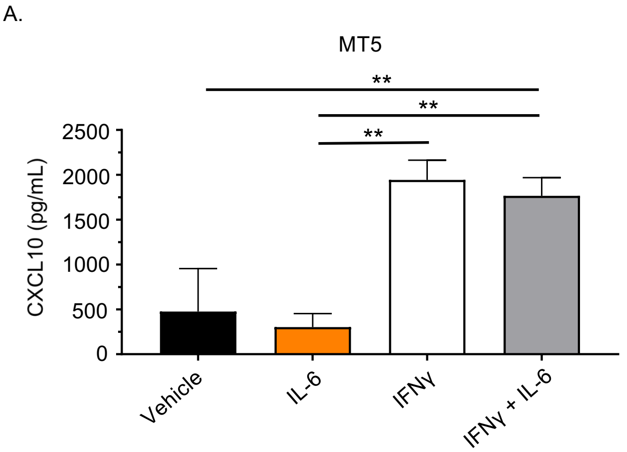

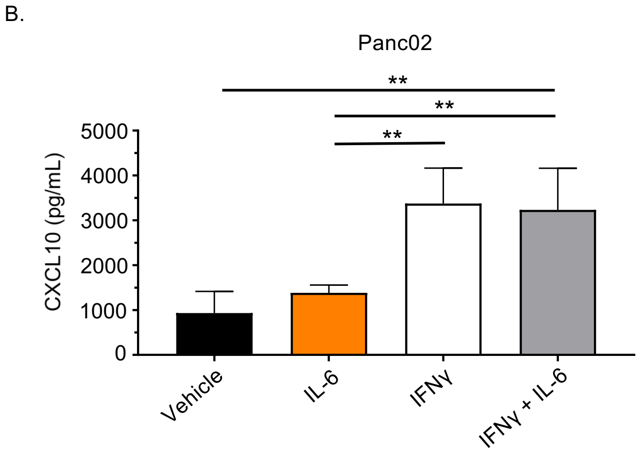

Supplemental Figure 4. ELISA for production of CXCL10 by murine **(A)** MT5 and **(B)** Panc02 PDAC cells stimulated with vehicle or treated with 10ng/ml IFNγ, 10ng/ml IL-6, or the combination of IFNγ and IL-6. Data is graphed as mean of 3 replicates ± SD, $^{**}p<0.01$.

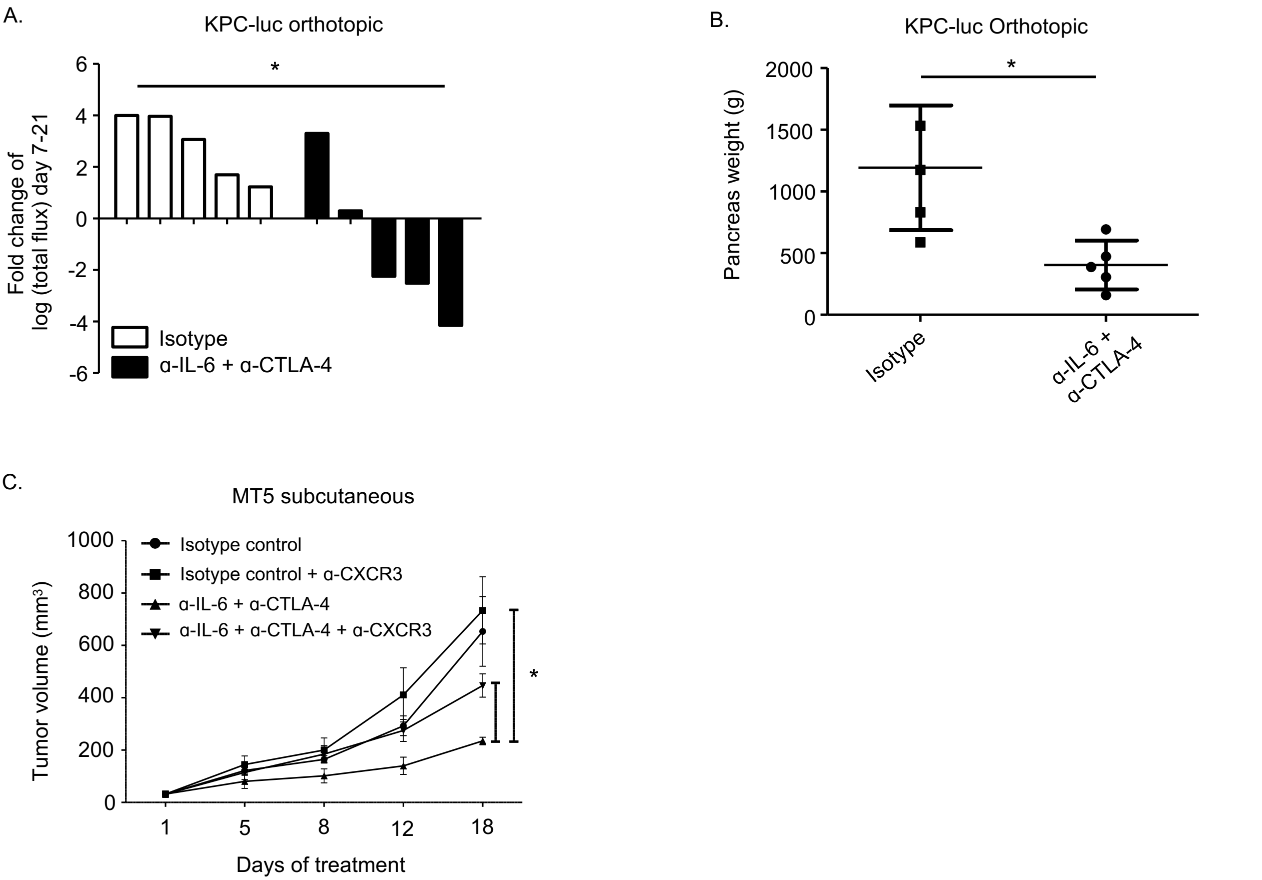

Supplemental Figure 5. 6-8wk old female C57BL/6 mice were orthotopically injected with 2 x 10^5 KPC-luc cells and treated with blocking antibodies to IL-6 and CTLA-4 or Isotype controls as previously described. **(A)** Mice were imaged weekly by bioluminescent imaging and the fold change in log of total flux was calculated. Students unpaired one-tailed t-test was used to compare change between groups, *$p<0.05$ **(B)** At study end, pancreas containing the tumors were resected and weighed. Average pancreas weight within each group is shown. Students unpaired one-tailed t-test was used to compare change between groups, *$p<0.05$ **(C)** Tumor volumes from mice with subcutaneous MT5 tumors are graphed. Mice were treated with isotype control antibodies with or without CXCR3 blocking antibodies or blocking antibodies to IL-6 and CTLA-4 with or without CXCR3 blocking antibodies. Tumors were measured by caliper every 4 to 5 days. Tumor volumes were analyzed by one-way ANOVA to determine if tumor volumes were significantly different followed by unpaired students t-test for pairwise comparisons.

A.

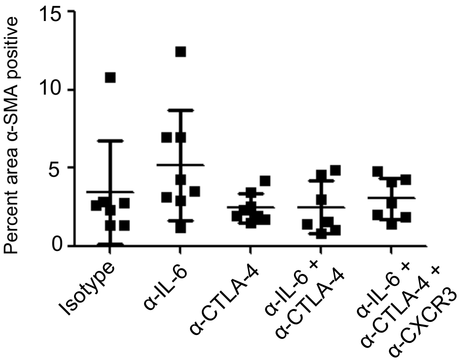

B.

Representative staining of α-SMA in FFPE tumors

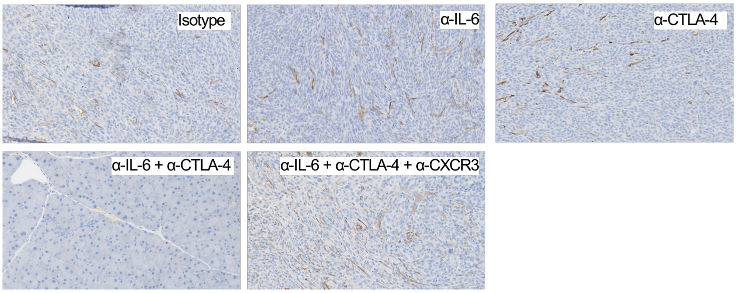

C.

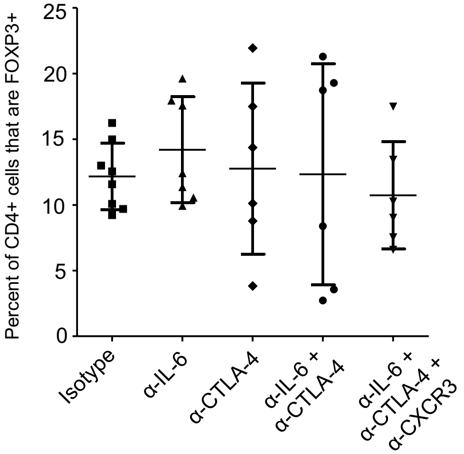

Supplemental Figure 6. Tissue slices of FFPE tumors from mice in the orthotopic study outlined in Figure 5 were stained for alpha-smooth muscle actin (αSMA) or CD4, FOXP3 and DAPI. **(A)** Each tissue slice stained for α-SMA was sampled based on total tissue area and the resulting 20x images were analyzed using FIJI to determine the percent area positive for α-SMA. The mean ± SD was then graphed for each treatment group. **(B)** Representative 20x images of α-SMA staining of tumors from each treatment group. **(C)** Immunofluorescent whole slide scans were collected on the Vectra Polaris Slide Scanner and analyzed using Qupath to determine the percentage of $CD4^{+}$ cells expressing FOXP3. The mean ± SD for each group was graphed.

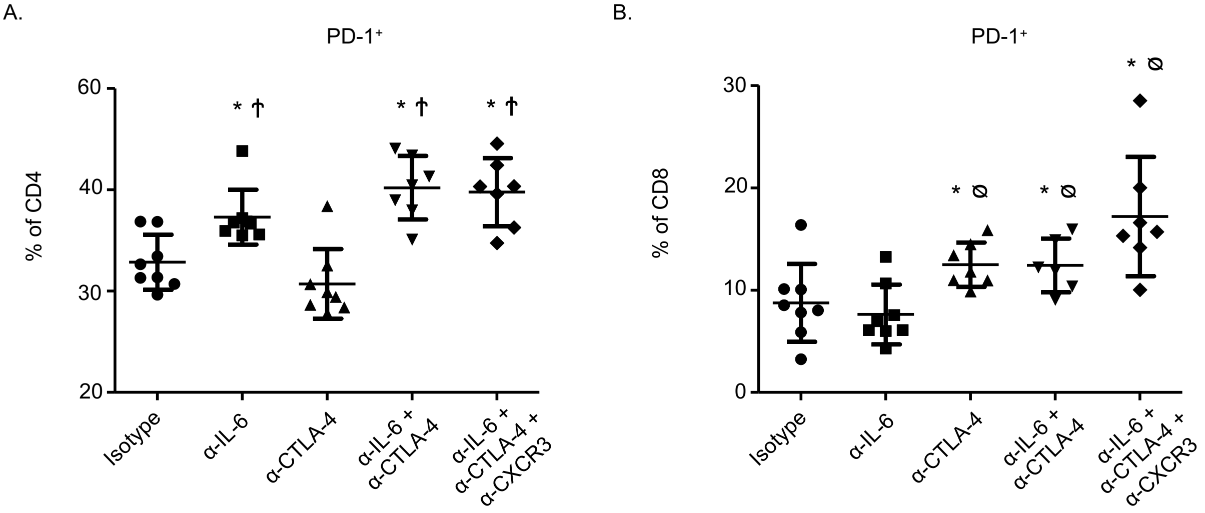

Supplemental Figure 7. Splenocytes isolated from mice treated in the orthotopic study described in Figure 5 were stained and the percentage of **(A)** CD4 T-cells expressing PD-1 or **(B)** CD8 T-cells expressing PD-1 were determined by flow cytometry analysis. Graphs show mean ± SD for each treatment group. * indicates significance ($p<.05$) to isotype control treated mice, ø indicates significance ($p<.05$) to mice treated with IL-6 antibody, and † indicates significance ($p<.05$) to CTLA-4 antibody treated mice.

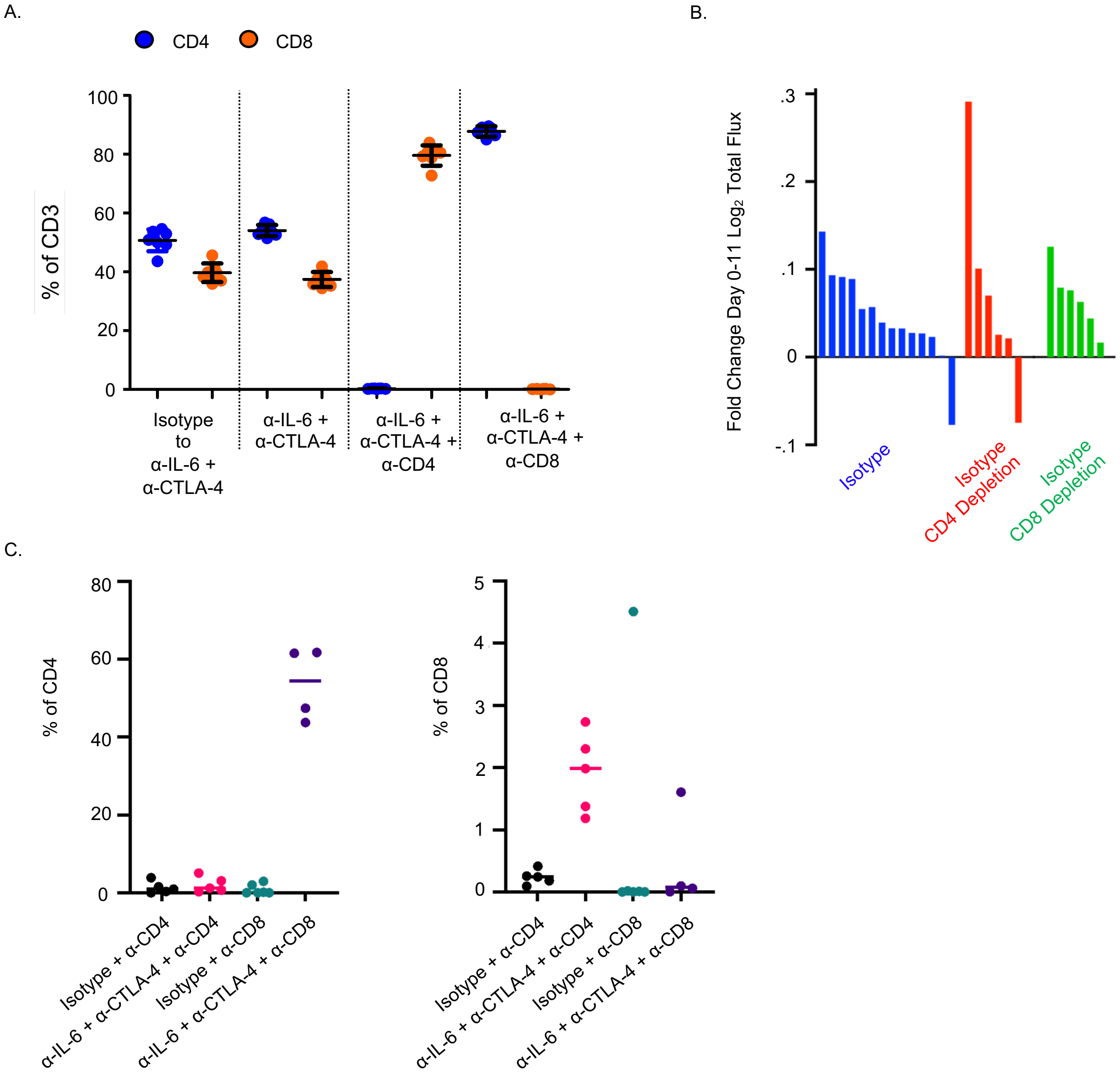

Supplemental Figure 8. **(A)** At the study endpoint from Figure 7, mice were euthanized and spleens were collected and processed for isolation of splenocytes. Splenocytes were then stained for CD3, CD4 and CD8 markers. The graph demonstrates the percentage of $CD3^+$ cells in splenocytes from each group that expressed the markers CD4 (blue) or CD8 (orange). **(B)** Tumor growth for each control group mouse from the study outline in **Figure 7A** was measured over time by BLI and the fold change in Log^2 of total flux for each mouse was graphed as a bar. **(C)** Flow cytometry was conducted on splenocytes from representative mice in each group to verify depletion of CD4 or CD8

Supplementary Table 1			
Antibody	Fluorophore	Clone	Source
CD11b	APC	M1/70	BD Biosciences
Ly6G	FITC	1A8	BD Biosciences
Ly6C	PE	AL-21	BD Biosciences
CD4	PE-Cy7	RM4-5	BD Biosciences
CD8	PE-Cy7	53-6.7	BD Biosciences
CD62L	PE	MEL-14	BD Biosciences
CD44	FITC	IM7	BioLegend
CXCR3	PE-Cy7	CXCR13-173	BioLegend
CCR4	PE	2G12	BioLegend
CCR6	APC	CK4-L3	BD Biosciences
RORγt	FITC	AFKJs-9	eBiosceince
CD3	Brilliant Violet 650	17A2	BioLegend
CD4	Brilliant Violet 711	GK1.5	BioLegend
CD8	Brilliant Violet 480	53-6.7	BD Biosciences
ICOS	Brilliant Violet 510	C398.4A	BioLegend
PD1	Brilliant Violet 421	RMP1-30	BioLegend
CD25	Alexa Fluor 488	PC61	BioLegend
CXCR3	PerCP/Cy5.5	CXCR3-173	BioLegend
CCR4	PE/Cy7	2G12	BioLegend
GATA3	PE-CF594	L50-823	BD Biosciences
RORyt	PE	Q31-378	BD Biosciences
CCR6	Alexa Fluor 647	29-2L17	BioLegend
FOXP3	Alexa Fluor 700	FJK-16s	ThermoFisher
TBET	APC	4B10	BioLegend
CTLA4	BV605	UC10-4B9	BioLegend
CD44	FITC	IM7	BioLegend
TCF1/7	PE	7F11A10	BioLegend
TIM3	PECy7	RMT3-23	BioLegend
CD69	PerCP/Cy5.5	H1.2F3	BioLegend
CD39	Aleca Fluor 647	Duha59	BioLegend
CD11b	BV510	M1/70	BioLegend
I-A/I-E	BV650	M5/114.15.2	BioLegend
CD206	BV711	C068C2	BioLegend
CD24	BV605	M1/69	BioLegend
B7-1	BV421	16-10A1	BioLegend
Ly6G	FITC	1A8	BioLegend
CD11c	Alexa Fluor 532	N418	ThermoFisher
Ly6C	PE-Cy7	Al-21	BD Biosciences
F4/80	PE	BM8	BioLegend
B7-2	PerCp/Cy5.5	GL-1	BioLegend
CD103	APC	2E7	BioLegend
CD64	Alexa Fluor 647	X54-5/7.1	BioLegend
VB14	FITC	14-2	BD Biosciences
CD4	PE	RM4-5	BD Biosciences
IFN-γ	BV450	XMG1.2	BioLegend
Live/Dead		Zombie Aqua Fixable Viability Kit	BioLegend
Rat IgG2b, k Isotype Ctrl Antibody	BV421	RTK4530	BioLegend
Mouse IgG1, k Isotype Control	PE-CF594	X40	BD Biosciences
Rat IgG2a Kappa Isotype Control	Alexa Fluor 700	eBR2a	ThermoFisher
Mouse IgG1, k Isotype Ctrl Antibody	APC	MOPC-21	BioLegend
Rat IgM, k Isotype Control	PE-Cy7	R4-22	BD Biosciences
Rat IgG2a, k Isotype Ctrl Antibody	PerCP/Cy5.5	RTK2758	BioLegend
CD4	Unconjugated	EPR19514	abcam
CD8	Unconjugated	Catalog Number ab203035 - polyclonal	abcam
FOXP3	Unconjugated	Catalog Number NB100-39002 - polyclonal	Novus
CD3	Unconjugated	Catalog Number A0452 - polyclonal	Dako
CXCR3	Unconjugated	Catalog Number BS-2209R	Bioss

Supplementary Table 2. P-values for flow cytometric analysis of splenocytes from mice treated with antibodies to IL-6, CTLA-4, the combination, or isotype controls.				
	TBET	GATA3	RORγt	Tregs
Isotype vs αIL-6+αCTLA-4	0.0015	<0.0001	0.1591	0.22
Isotype vs αIL-6	0.0041	0.7235	0.0088	0.518
Isotype vs αCTLA-4	0.1142	0.0009	0.3816	0.1146
Isotype vs αIL-6+αCTLA-4+αCXCR3	0.0005	<0.0001	0.0256	0.6526
αIL-6 vs αCTLA-4	0.0001	0.0002	0.0001	0.1927
αIL-6 vs αIL-6+αCTLA-4	0.5765	0.0003	0.1493	0.8565
αIL-6 vs αIL-6+αCTLA-4+αCXCR3	0.402	<0.0001	0.5205	0.7476
αCTLA-4 vs αIL-6+αCTLA-4	<0.0001	0.0012	0.0094	0.0251
αCTLA-4 vs αIL-6+αCTLA-4+αCXCR3	<0.0001	<0.0001	0.0003	0.1227
αIL-6+αCTLA-4 vs αIL-6+αCTLA-4+ αCXCR3	0.8097	0.7521	0.3471	0.4841
